# Editorial: Premalignant conditions in the esophagus and stomach

**DOI:** 10.3389/fonc.2022.1091911

**Published:** 2022-11-28

**Authors:** Francisco Tustumi, Diogo Turiani Hourneaux de Moura, Jaques Waisberg, Fernando Augusto Mardiros Herbella

**Affiliations:** ^1^ Universidade de São Paulo, Department of Gastroenterology, Sao Paulo, Brazil; ^2^ Hospital Israelita Albert Einstein, Department of Surgery, Sao Paulo, Brazil; ^3^ Centro Universitário Faculdade de Medicina do ABC, Department of Surgery, Santo Andre, Brazil; ^4^ Universidade Federal de São Paulo, Department of Surgery, Sao Paulo, Brazil

**Keywords:** esophageal neoplasm, gastric neoplasm, achalasia, Barrett esophagus, atrophic gastritis

Over the years, the development of minimally invasive procedures, endoscopy and chromoscopy, serum and histologic biomarkers, and epidemiology have allowed early detection, management, and treatment of premalignant lesions, conditions associated with high risk for cancer, and early neoplasms. The earliest management, investigation, and follow-up of premalignant lesions and superficial neoplasms in the esophagus and stomach are essential, as they can further improve the survival rates and reduce local and systemic recurrence by allowing prompt curative interventions.

## Understanding cancer initiation and progression

Several conditions promote a higher risk for the development of esophagogastric cancer. It encompasses Barrett’s esophagus, achalasia, atrophic gastritis, esophagitis of varying types (e.g., infectious, drug-induced, eosinophilic, etc.), esophageal stenosis due to ingestion of corrosive agents, polyposis, among others. Each condition promotes a different risk ratio for cancer development (1, 2).

**Figure 1:**
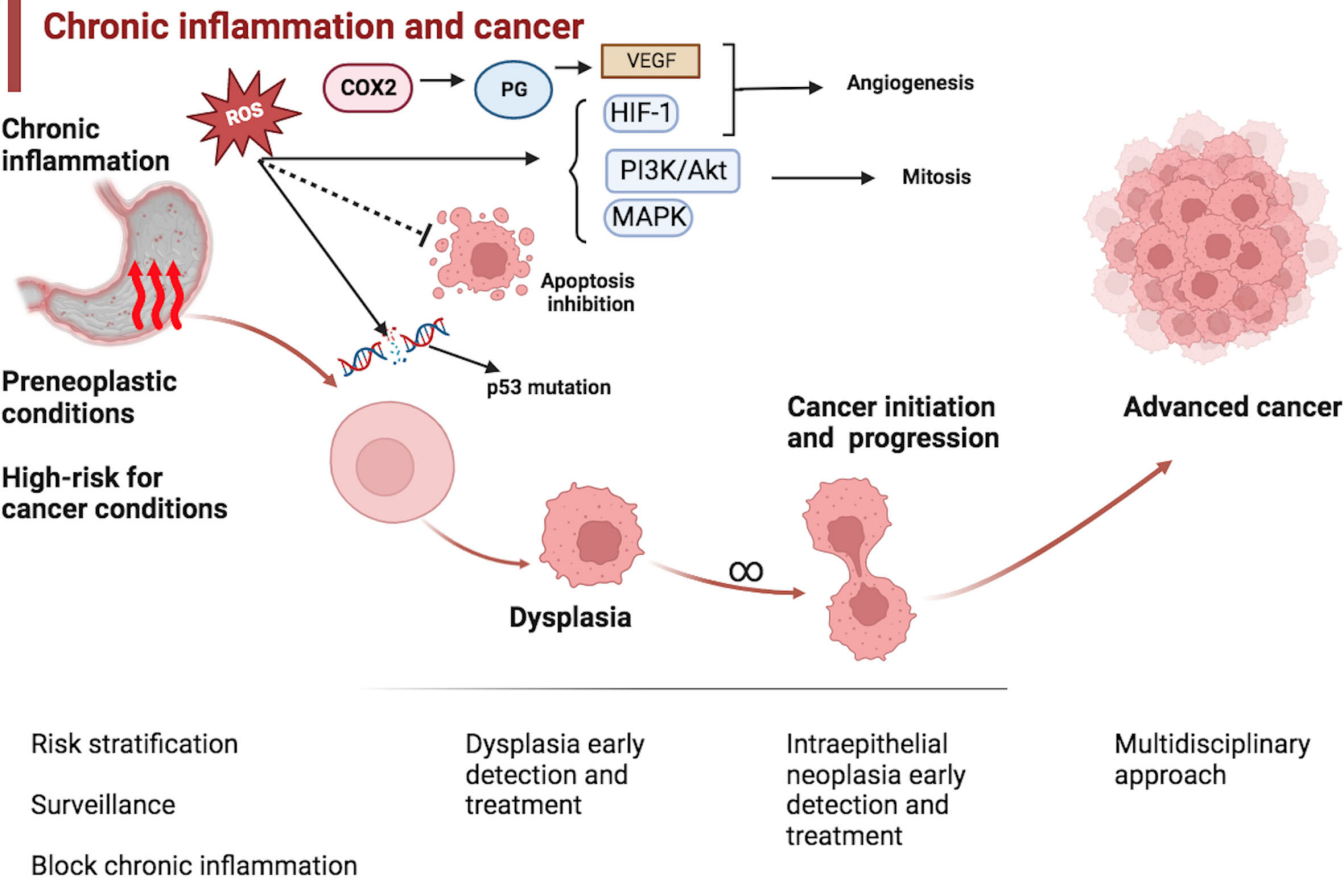
Chronic inflammation and esophagogastric cancer. Preneoplastic and high-risk cancer conditions are usually associated with the epithelium’s chronic inflammation. Chronic inflammation may promote oncogenesis through several pathways, including DNA damage, chromosomal instability, and mutations. Reactive oxygen species (ROS) inhibit apoptosis, and promote angiogenesis and proliferation. Clinicians should be ready to act at every stage of cancer initiation and progression, including blocking chronic inflammation, surveillance, risk stratification, dysplasia and early cancer eradication, and advanced cancer management. COX2: Cyclooxygenase-2; PG: Prostaglandin; VEGF: Vascular endothelial growth factor; HIF-1: Hypoxia-inducible factor 1; PI3K/Akt: Phosphoinositide 3-kinases/Protein kinase B; MAPK: Mitogen-activated protein kinase.

Chronic inflammation is a factor that is common for several of these conditions (3). Chronic inflammation may contribute to oncogenesis through several different pathways, including DNA damage, angiogenesis, and deregulation of cellular growth, proliferation, and death (4). See **Figure 1**.

By blocking the underlying mechanism for cancer initiation and progression, clinicians could propose preventive measures.

## Prevention measures

Inhibition of chronic aggression to the esophagogastric epithelium and avoiding tissue inflammation is the first measure clinicians should consider in premalignant conditions. Despite previously published studies showing conflicting findings, theoretically, blocking gastroesophageal reflux, handling achalasia outflow obstruction, and treating infections or underlying autoimmune conditions is the first step for preventing cancer initiation and progression (5–7). *H. pylori* eradication is a controversial issue, although chronic gastric inflammation due to this infection may be associated with ulcers and some gastric adenocarcinoma and lymphoid tissue lymphoma (8).

Some studies’ protocols have evaluated the role of cyclooxygenase (COX) enzyme inhibition as chemoprophylaxis for the adenoma-adenocarcinoma sequence. The inhibition of COX-2, extensively present in the gastrointestinal tract, reduces prostaglandin E2 production, downregulating inflammation oncogenesis pathways (9). COX inhibitors, such as aspirin, could have a significant role in inherent conditions, such as familial adenomatous polyposis (10, 11). However, most of the current knowledge is focused on colorectal polyps, and few are debated for those located in the upper gastrointestinal system. Future trials are still needed for definitive answers.

## Stratifying risks

Life-expectation estimation is the first point to be considered when evaluating the risk for progression to dysplasia or cancer. How long will your patient live? How long would it take for the cancer development? This information should be estimated based on the patient’s clinical status, age, comorbidities, and average life expectations for the patient population. If you judge that the probability of developing cancer is extremely low along a patient’s lifespan, there would be no reason for aggressive surveillance programs or interventions.

Risk variables are usually evaluated in regression models of cohort studies. Several tools may facilitate routine clinical risk estimation in clinical practice. Nomograms and risk calculators facilitate daily practice risk stratification. In a Chinese retrospective cohort, Sun et al. evaluated the risk of chronic atrophic gastritis progression to intraepithelial neoplasia. The authors evaluated staging systems for classifying the gastritis severity “operative link on gastritis assessment” (OLGA) and “operative link on gastric intestinal metaplasia assessment” (OLGIM) and found that both were closely associated with progression to intraepithelial neoplasia.

## Surveillance and early detection

Patients with premalignant conditions should not have similar screening strategies to the healthy population. These patients should be under a close surveillance program to allow early detection and treatment at diseases’ early stages. Surveillance should be individualized according to the stratified risk estimation.

In the esophagus and stomach, endoscopies are the most relevant tests for surveillance. Chromoendoscopy may help detect early lesions. Virtual chromoendoscopy, such as narrow banding imaging (NBI), highlights the mucosa surface and vascular pattern. NBI and Lugol’s solution chromoendoscopy have similar sensitivity for detecting suspicious lesions, but NBI has higher specificity (12, 13). Other helpful systems include Pentax iScan and Fujinon Intelligent Color Enhancement (FICE). Volumetric laser chromoendoscopy uses optical coherence tomography with infrared light. This technology provides many cross-sectional images, and future artificial intelligence studies may help process these images (14, 15). Sequential sampling biopsies, such as the Seattle protocol, and wide-area transepithelial sampling (WATS) may also help detect dysplasia and early cancer (16, 17).

## Management

Grading of dysplasia, evaluating risk for nodal involvement, and mucosal abnormalities findings help determine surgical or locoregional therapy. Dysplasia progression usually demands endoscopic eradication therapy, such as endoscopic mucosal resection, radiofrequency ablation, cryotherapy, and hybrid argon plasma coagulation. Endoscopic mucosal or submucosal resection has the advantage of allowing pathological evaluation and risk stratification. Visible mucosal abnormalities should be staged by endoscopic resection, which could also work as eradication therapy (18).

Endoscopic resection is usually the preferred method for early cancer when there is no suspicion of lymph node metastasis. Bestetti et al., in a systematic review and meta-analysis, compared endoscopic resection with surgery for early gastric cancer. The authors found that endoscopic resection was associated with lower adverse events and shorter lengths of stay than surgery, with similar long-term survival rates.

Surgical resection should be considered for dysplasia progression or early cancer in some end-stages conditions, such as sigmoid megaesophagus and severe strictures. Particular attention should be taken to the cancer development in stenosis due to ingestion of corrosive agents. Andreollo et al. reviewed the cancer dilemma in caustic stenosis. Cancer may develop in intramural fibrosis, and advanced cancer may be easily misinterpreted as early cancer. Often, cancer manifests in these patients as exophytic homogeneous enhancement in the esophageal wall or enlarged mediastinal lymph nodes.

Finally, any advanced cancer developing under an esophagogastric premalignant condition should be evaluated carefully with a multidisciplinary team comprising oncologists, radiotherapeutics, surgeons, nutrition, and palliative care.

## Conclusion

This Research Topic covers the role of premalignant and high-risk conditions in developing esophageal and gastric cancer. Understanding cancer initiation and progression, knowing prevention measures, stratifying risks, screening, and proper management are key issues that the studies published along this Topic could approach.

## Author contributions

All authors listed have made a substantial, direct, and intellectual contribution to the work and approved it for publication.

## Conflict of interest

The authors declare that the research was conducted in the absence of any commercial or financial relationships that could be construed as a potential conflict of interest.

## Publisher’s note

All claims expressed in this article are solely those of the authors and do not necessarily represent those of their affiliated organizations, or those of the publisher, the editors and the reviewers. Any product that may be evaluated in this article, or claim that may be made by its manufacturer, is not guaranteed or endorsed by the publisher.
